# Mother-child pairs’ eating and feeding behaviours in two different nutritional status from two distinct provinces

**DOI:** 10.1186/s12887-023-04481-5

**Published:** 2024-01-08

**Authors:** Gülsüm Özen, Bülent Güneş, Suzan Yalçın, Sıddika Songül Yalçın

**Affiliations:** 1 Ankara Ataturk Sanatoryum Training and Research Hospital, Keçioren, Ankara Turkey; 2 Child Health and Disease Service, Şanlıurfa Training and Research Hospital, Şanlıurfa, Turkey; 3 Department of Food Hygiene and Technology, Selcuk University Faculty of Veterinary Medicine, Konya, Turkey; 4 Department of Pediatrics, Hacettepe University Faculty of Medicine, Ankara, Turkey

**Keywords:** Thinness, Feeding problems, Eating behaviours

## Abstract

Nutrition plays a crucial role for the bio-psycho-social growth and development of children. Mother-child pairs’ eating-feeding problems can be hypothesized to relate to the child’s nutritional status and living area. Our focus was to assess maternal attitudes and children’s eating habits in thin and normal-weight children across two cities characterized by distinct socioeconomic and demographic profiles: Ankara and Şanlıurfa. This double case-control study included the mothers whose children are aged between 2 and 7 years old in both cities. Figure Rating Scale, Child Feeding Questionnaire and Behavioral Pediatrics Feeding Assessment Scale were filled by mothers of children. The mothers of thin children were concerned more about the weight of their children, felt more responsible for feeding their children, and use of pressured them to eat more frequently compared to normal-weight children’s mothers in both cities. In Ankara, the duration of breastfeeding is longer, using formula is rarer and starting complementary food on time is more common, mother had higher scores on perceived responsibility, perceived child weight, and concern about child weight subscales; lower score on the restriction subscale compared to Şanlıurfa. Mothers of both thin and normal weight children in Şanlıurfa rated their children as weaker compared to those in Ankara. In conclusion, child eating behaviors and parental feeding practices were associated with the weight of children depending on demographic and sociocultural differences. Implementing customized, location-specific preventive educational initiatives on child feeding will not only foster behavioral changes but also yield valuable insights for shaping future interventions.

**Supplementary Information:**

The online version contains supplementary material available at 10.1186/s12887-023-04481-5.

## Introduction

Correct and adequate nutrition in infancy and preschool period is of great importance for the bio-psycho-social growth and development of children [1]. Children’s attitudes about what, where, when, and how much to eat are shaped by social and environmental factors such as eating and feeding attitudes, habits, beliefs of families, and what kind of foods are available at home [2]. Child feeding behaviors of families are important especially in the toddler and preschool age groups due to the critical role of the habits gained in this period both support growth and development and determine the eating behaviors in the later period of life [3].

Geographic and ethnic differences can also cause changes in the eating habits of both parents and children, and in the feeding behavior of parents [4, 5]. Similarly, differences in food choices are seen even in different regions of Turkey as well [5]. While more rice is eaten in the Aegean and Marmara regions, bulgur is consumed much more in Anatolia. In the south and southeast Anatolian region, we encounter many dishes in common with Syrian and Iranian cuisine. The habit of eating various salads, meatballs, and hot peppers in meals in these regions is also seen as a result of this interaction [6]. Nutrition habits also differ according to the socio-economic levels of the families [5, 7, 8]. That is, the higher the income, the higher the level of food consumption. For example, in a nationwide study in Turkey, as income increases, consumption of bread and other wheat products decreases, while rice consumption increases. Foodstuffs rich in carbohydrates (grain) are consumed in sufficient quantities without much difference between low and higher-income families. On the other hand, the fact that protein consumption, especially animal protein consumption, is far below normal in low-income families also causes unbalanced nutrition [7, 8].The city with the highest total fertility rate in Turkey was Şanlıurfa with 3.81 children [9]. Compared to Ankara, younger maternal age, having more children, lower education, and lower-income status were reported in Şanlıurfa [10]. These two cities, which have socio-cultural and economic diversities, may also differ in the eating habits of children and the feeding behaviors of their mothers.

It has been shown that mother-child pairs’ behaviors and styles that are specific to the feeding context also affect children’s anthropometry [11–13]. There might be an interaction between characteristics of living areas and mother-child pairs’s eating-feeding [14]. There are some questionnaires that evaluate feeding characteristics [15–17]. Behavioral pediatric feeding assessment scale (BPFAS) which is one of the most reliable, standardised tool validated in many languages and is used safely in more than 20 countries, was prepared to determine child’s pathologic eating behavior [17–19]. The child feeding questionnaire (CFQ) was a tool that evaluates parents’ perceptions, concerns and practices related to child feeding, as well as their children’s development of an eating pattern with these behaviors and the relationship between obesity and control of food intake and the information was provided by the parents of children aged 2 to 11 years [13]. It is widely used in many countries around the World [15, 16, 20]. However, no published study is present about mother-child pairs’ eating-feeding problems according to the child nutritional status and living area.

In the theoretical framework, children’s eating behaviors are partly biologically based, but the behaviors change or develop with environmental influences [21]. We hypothesize that there is an association between parenting style, feeding practices, and child nutritional status.In this study, the purpose is to evaluate maternal attitudes in the feeding process, and eating habits of children aged 2–7 in thin and normal-weight children from two cities having distinctive socioeconomic and demographic characteristics. Thus, regional programs that will support the healthy nutritional status of children can be created and measures can be taken in the early period against the factors restricting growth and development with the results of the study.

## Material method

This double case-control study included 408 mothers whose children are aged between 2 and 7 years old and admitted for child health supervision to Hacettepe University İhsan Dogramacı Hospital in Ankara and Şanlıurfa Training and Research Hospital in Şanlıurfa between November 2022- January 2023. All voluntary mothers having children aged between 2 and 7 years old who meet the inclusion criteria were included in the study.

The sample size for 2 (thin and normal-weight) independent groups in one city was calculated as effect size (f, medium conventions): 0.25, alpha error: 0.05, power: 0.95 was 210 (G*Power 3.1.9.4). And for two cities 420 was planned.

The ethics committee approved the consent procedure. This clinical study was approved by the Harran University Regional Ethics Board (HRU/22.21.21) and was conducted according to the Declaration of Helsinki. After giving information about the aim and design of the study, the parents provided written informed consent on behalf of the children enrolled in the study.

### Data collection

The children who have chronic diseases, younger than 2, older than 7 year old and the parents who refused to participate in the study were not taken for the study. For each underweight child, normal-weight children of the same age and sex who came to the outpatient clinic were included.

The study included four forms filled by parents: sociodemographic data form, Figure Rating Scale [22], CFQ [11, 20, 23], and BPFAS [14, 18]. The forms were read and marked by authors for illiterate parents. The sociodemographic data form included questions for the mother’s age, education level, occupation, history of illness during pregnancy of mothers, number of children, family structure, income level, city of residence, enrolled child’s age, gender, height, weight, birth weight and type of delivery, history of breast-feeding and complementary feeding. CFQ and BPFAS are self-reported measurement tools. They measure subjective data but include several subscales where the results support each other.

Children’s height and weight were measured and noted on the day of their examination. Body mass index (BMI) was calculated from the weight and height (the formula is calculated as weight in kilograms divided by height in meters squared, BMI = kg/m^2^) of each child during child health surveillance. According to WHO, BMI < 5 percentile was underweight, 5–14th percentile: thin, 15th to 84th percentile: healthy weight; 85–94th percentile: overweight, ≥ 95th percentile: obese) [24]. Therefore, thin group was defined as an age- and sex-specific BMI between 5 and 14th percentiles; healthy weight group was accepted as between 15 and 75th percentile in our study.

### Measures

**Figure Rating Scale** was developed by Collins in 1991 to examine perceptions of body figures. It consists of seven female/male images ranging from 1 (underweight) to 7 (obese) on this Likert scale [22]. Mothers were asked to identify the image of their children that looked most similar and the ideal figure that they desired for their children. Results showed the child’s perceived and desired body image by the mother.

**Child Feeding Questionnaire** (CFQ) was developed as 24 items [13]. This caregiver-reported tool was revised and the number of items was increased to 31 [25]. Items scored between 1 and 5 on a Likert-type scale. The revised CFQ consists of 7 sub-dimensions. Four sub-dimensions (Perceived Responsibility, Perceived Parent Weight, Perceived Child Weight, and Concern about Child Weight) measure aspects of parents’ perceptions and concerns about their children’s risk for obesity, while the other three sub-dimensions (Restriction, Pressure to eat, Monitoring) assess parents’ use of controlling feeding practices. The scale does not have a total score, each sub-dimension is scored on its own. Increasing scores for each sub-dimension indicate the strength of parents’ attitudes toward that behavioral model. Erdim et al. performed Turkish validation of CFQ [23].Since our study did not include children over the age of 7, questions 11, 12, 13 were not taken for the study. Cronbach alpha values for Perceived Responsibility, Perceived Parent Weight, Pressure to eat, and Monitoring were similar in our study (Table 1) to the validation study [23] Perceived Parent Weight, Concern about Child Weight, Pressure to eat subscales have higher Cronbach alpha values in healthy growth children than thin children group. Besides, Restriction subscales have lower Cronbach alpha values in healthy-typical growth children (Table 1).

**Table 1:** Distribution of scores of Child Feeding Questionnaire and Behavioral Pediatrics Feeding Assessment Scale and Cronbach alpha values

					Percentiles					Item, n	Cronbach alpha values					
	Mean	SD	Min.	Max.	10	25	50	75	90		Study: [23]	Our study	Ankara	Şanlıurfa	Thin group	Normal weight group
**Child Feeding Questionnaire scales**																
Perceived Responsibility	4.1	0.8	2.0	5.0	3.0	3.7	4.0	5.0	5.0	3	0.59	0.51	0.66	0.41	0.52	0.49
Perceived Parent Weight	2.9	0.6	1.0	5.0	2.0	2.5	3.0	3.3	3.8	4	0.70	0.74	0.73	0.76	0.68	0.78
Perceived Child Weight	1.6	0.3	0.6	2.6	1.2	1.4	1.6	1.8	2.0	3	0.77	0.69	0.65	0.68	0.64	0.64
Concern about Child Weight	3.7	0.7	1.3	5.0	2.7	3.3	3.7	4.3	4.7	3	0.72	0.46	0.55	0.41	0.46	0.41
Restriction	3.8	0.7	1.4	5.0	2.9	3.4	3.9	4.4	4.8	8	0.79	0.56	0.57	0.55	0.61	0.50
Pressure to Eat	4.0	1.0	1.0	5.0	2.5	3.5	4.0	4.8	5.0	4	0.70	0.65	0.70	0.61	0.43	0.70
Monitoring	4.0	0.9	1.0	5.0	3.0	3.3	4.0	5.0	5.0	3	0.81	0.85	0.85	0.87	0.87	0.83
**Behavioral Pediatrics Feeding** **Assessment scales**											Study: [17]					
Picky eaters	19.2	5.4	9.0	34.0	12.9	15.0	19.0	23.0	27.0	7	0.76	0.70	0.76	0.65	0.69	0.64
Toddler general refusal	12.0	4.3	5.0	24.0	6.0	9.0	12.0	15.0	18.0	5	0.74	0.69	0.70	0.66	0.66	0.71
Toddler textured food refusal	7.0	2.6	5.0	21.0	5.0	5.0	6.0	8.0	10.0	5	0.72	0.54	0.63	0.42	0.57	0.45
Older child refusal	14.9	4.4	6.0	27.0	9.0	12.0	15.0	18.0	21.0	7	0.77	0.54	0.58	0.50	0.46	0.57
Total BPFAS	53.1	13.0	26.0	85.0	36.0	43.0	52.5	62.0	71.0	24	0.88	0.84	0.87	0.80	0.81	0.83

n = number, SD = standard deviation, min = minimum, max = maximum

**Behavioral Pediatrics Feeding Assessment Scale** (BPFAS) is a parent-answered scale used to determine eating behavior in pediatrics [26]. The scale is a 5-point Likert type (1 = Never, 2 = Rarely, 3 = Sometimes, 4 = Frequently 5 = Always) and consists of 35 items and 5 sub-dimensions; 25 of the statements are about the nutritional status of the child, and 10 of them about the person who is responsible for feeding a child. The Turkish adaptation study covers only 4 sub-dimensions and 24 items related to the nutritional status of the child; picky eaters (7 items), toddler refusal- general (5 items), toddler refusal- textured food (5 items), older child refusal (7 items) [17]. Seven statements in the scale (items 1, 3, 5, 6, 8, 9, and 16) convey positive connotations, while 18 of them carry negative meanings. Notably, positive items are scored inversely. A rise in the overall score from the scale indicates a higher level of problematic eating behaviors and habits. Cronbach alpha was found 0.88 for the 4 sub-dimensions of BPFAS (Picky eaters: 0.76, toddler general refusal: 0.74, toddler textured food refusal: 0.72, older child refusal: 0.77). Cronbach alpha values for picky eaters and toddler general refusal subscales were similar in our study (Table 1) to the previous adaptation study [17] However, other subscales; “Toddler textured food refusal and Older child refusal” showed lower Cronbach alpha values than the previous study [17]. “Toddler general refusal” and “older child refusal” subscales have higher Cronbach alpha values in healthy growth children than thin children group. Besides, picky eaters and toddler textured food refusal subscales have lower Cronbach alpha values in healthy growth children (Table 1). While the highest Cronbach alpha value was in the toddler general refusal group with healthy body weight, the lowest Cronbach alpha value was in the toddler textured food refusal group with healthy body weight. A reliability coefficient above 0.70 indicates that the scale is reliable [17].

**Statistical analysis** was performed using IBM Statistical Package for Social Sciences (IBM-SPSS Statistics 23, IBM Inc, Chicago, IL, USA). Histogram, Skewness, and Kurtosis values were used in addition to Kolmogorov-Smirnov test for normality distribution. Chi‐square was used to compare categorical groups. Independent samples t‐test was used to compare the averages of two independent groups with normal distribution and Mann–Whitney U test was used to compare the median of two independent groups with no normal distribution.

Subscales of CFQ including Perceived responsibility, Parental Concern about Child Weight, Restriction, Pressuring Children to Eat More, and Image of desired weight of child were left skewed. Histograms of Perceived Parent Weight and Perceived Child Weight showed normal distribution. All subscales of BPFAS scores and Images of perceived weight of the child showed right-skewed.

Subscales’ scores of CFQ, BPFAS, and Figure Rating Scale in distinct cities and nutritional status were compared with interactions with generalized linear models (Model 1), and estimated mean [95% CI] were given. In further analysis, generalized linear model-2 performed the association between a score of each scale (CFQ, BPFAS, and Figure Rating Scale) and groups (study center and nutritional status) with confounding factors [Mother’s age (years), mother’s education (Illiterate, primary-middle, high school, university), income (low, middle), child number (n), birth weight (g)].

The significance level was accepted if the p-value was less than 0.05 (P < 0.05).

## Results

In total, 408 mothers and child pairs were included in this study, 204 of them were from Ankara and others were from Şanlıurfa (Table 2). The BMI percentiles of 200 children were between 5 and 15 % from both cities. The other 208 children had a BMI in the normal healthy range (25–75th percentiles).

**Table 2:** General characteristics of the mother-child pairs according to nutritional status and study center

n		Nutritional status			Study center		
	Overall	Thin	Normal	p	Ankara	Şanlıurfa	p
	408	200	208		204	204	
**Mother’s age, years**	31.2 ± 5.4	31.8 ± 5.5	30.6 ± 5.3	0.028	31.9 ± 5.3	30.5 ± 5.4	0.009
**Mother’s education**				0.220			< 0.001
Illiterate, primary incomplete	9.8	10.0	9.6		5.9^a^	13.7^b^	
Primary-Middle	57.6	62.0	53.4		42.2^a^	73.0^b^	
High school	20.3	18.5	22.1		31.9^a^	8.8^b^	
University	12.3	9.5	14.9		20.1^a^	4.4^b^	
**Disease in pregnancy**	38.5	39.5	37.5	0.678	37.7	39.2	0.760
**Hypertension**	6.9	7.5	6.3	0.618	6.9	6.9	1.000
**Diabetes mellitus**	6.4	7.0	5.8	0.611	7.8	4.9	0.224
**Iron deficiency**	19.9	20.0	19.7	0.942	15.2	24.5	0.018
**Vitamin B12 deficiency**	2.7	3.0	2.4	0.710	3.9	1.5	0.126
**Hypothyroidism**	3.4	2.0	4.8	0.119	5.9	1.0	0.007
**Risk of abortion history**	2.2	3.5	1.0	0.081	1.0	3.4	0.092
**Incomes, poor**	35.0	35.3	34.6	0.851	21.6	48.5	< 0.001
**Number of children**	2.8 ± 1.3	2.9 ± 1.4	2.6 ± 1.3	0.029	2.3 ± 1.0	3.2 ± 1.5	< 0.001
1	10.5	8.0	13.0	0.170	13.7^a^	7.4^b^	< 0.001
2–3	69.9	70.0	69.7		78.4^a^	61.3^b^	
≥ 4	19.6	22.0	17.3		7.8^a^	31.4^b^	
**Age of enrolled child, month**	51 ± 17	51 ± 17	50 ± 17	0.359	50 ± 17	52 ± 17	0.308
**Gender, male**	50.0	48.0	51.9	0.428	52.5	47.5	0.322
**Weight at birth, gram**	3144 ± 530	3105 ± 532	3152 ± 611	0.410	3171 ± 501	3088 ± 636	0.142
< 2500	6.6	7.5	5.8	0.747	1.0^a^	12.3^b^	< 0.001
2500–3999	88.0	87.5	88.5		94.1^a^	81.9^b^	
≥ 4000	5.4	5.0	5.8		4.9^a^	5.9^a^	
**Type of delivery, Cesarean**	48.8	48.0	49.5	0.759	49.5	48.0	0.766
**Duration of breastfeeding**				0.786			< 0.001
< 5 month	20.1	20.5	19.7		15.7^a^	24.5^b^	
6–11 month	17.4	17.0	17.8		13.2^a^	21.6^b^	
12–23 month	42.6	44.5	40.9		40.7^a^	44.6^a^	
≥ 23 month	19.9	18.0	21.6		30.4^a^	9.3^b^	
**History of formula milk use**	45.3	46.5	44.2	0.645	40.2	50.5	0.037
**Starting age for complementary food**				0.984			< 0.001
< 6 month	18.6	18.5	18.8		26.0^a^	11.3^b^	
6 month	64.2	64.0	64.4		71.1^a^	57.4^b^	
≥ 7–9 month	17.2	17.5	16.8		2.9^a^	31.4^b^	
**Meals on a day**	2.5 ± 1.5	2.3 ± 1.4	2.7 ± 1.5	0.002	2.9 ± 1.3	2.1 ± 1.5	< 0.001
< 3 times, insufficient	43.6	51.5	36.1	0.002	32.4	54.9	< 0.001

a,b: indicate differences in the same row for the same variable

*Values were given as mean ± standard deviation or column percentage

### General characteristics of groups

Mothers from Ankara Center and mothers having thin child were older than counterparts (p = 0.009, 0.028, respectively, Table 2).

A very large number of mothers from Şanlıurfa Center were illiterate or primary school graduates compared to Ankara Center (p < 0.001), about half of the participants in Şanlıurfa Center reported their income as low (p < 0.001). The total number of children in the family was found to be higher in Şanlıurfa Center and in thin child group than counterparts (p < 0.001, p = 0.029; respectively). The median age of children was 4.2 years (range: 24–83 months) and 50% were males. Both study groups had similar age and sex distribution.

The children living in Şanlıurfa Center had a higher percentage for low birth weight history (12.3 vs. 1.0%, respectively), shortened time of getting breastfeeding (< 5 months: 24.5 vs. 15.7%, and 6–11 mo: 21.6 vs. 13.2%, respectively) and delayed time to receive complementary food (≥ 7–9 month 31.4 vs. 2.9%, respectively) comparing to those in Ankara (p < 0.05, Table 2). While the use of formula milk was more common in Şanlıurfa, only 53.9% of them breastfed for more than 1 year, this rate was 71.1% in Ankara (p < 0.001). Children from Şanlıurfa center and thin group consumed more frequently insufficient meals on a day compared to counterparts (p < 0.001, p = 0.002; respectively).

### CFQ scores in groups

The scores of Perceived Parent Weight, Monitoring, and Restriction subscales did not change according to both study center and nutritional status (Table 3).

**Table 3:** Scores of Child Feeding Questionnaire (CFQ) and Behavioral Pediatrics Feeding Assessment Scale (BPFAS) and Figure rating scales (FRS) by weight of children and cities

Scales	Study Center (B)			Sign, p*			
Nutritional status (A)	Ankara	Şanlıurfa	Overall, A	A	B	AXB	
Child Feeding Questionnaire scores							
**Perceived Responsibility**							
Thin	4.41 [4.3–4.6]^a^	3.93 [3.8–4.1]^b^	4.17 [4.1–4.3]^x^	0.010	0.003	0.041	
Normal	4.09 [3.9–4.2]^b^	3.90 [3.8–4.1]^b^	4.00 [3.9–4.1]^y^				
Overall, B	4.25 [4.2–4.4]^m^	3.92 [3.8-4.0]^n^					
**Perceived Parent weight**							
Thin	2.85 [2.7-3.0]	2.93 [2.8-3.0]	2.89 [2.8-3.0]	0.095	0.686	0.749	
Normal	2.95 [2.8–3.1]	2.99 [2.9–3.1]	2.97 [2.9–3.1]				
Overall, B	2.90 [2.8-3.0]	2.96 [2.9-3.0]					
**Perceived Child Weight**							
Thin	1.49 [1.4–1.5]^b^	1.36 [1.3–1.4]^a^	1.43 [1.4–1.5]^x^	< 0.001	< 0.001	0.013	
Normal	1.84 [1.8–1.9]^d^	1.59 [1.5–1.6]^c^	1.72 [1.7–1.8]^y^				
Overall, B	1.67 [1.6–1.7]^m^	1.47 [1.4–1.5]^n^					
**Concern about Child Weight**							
Thin	4.00 [3.9–4.1]^a^	3.75 [3.6–3.9]^b^	3.87 [3.8-4.0]^x^	< 0.001	0.809	0.018	
Normal	3.49 [3.4–36]^c^	3.56 [3.4–3.7]^bc^	3.53 [3.4–3.6]^y^				
Overall, B	3.74 [3.6–3.8]	3.66 [3.6–3.8]					
**Restriction**							
Thin	3.73 [3.6–3.9]	3.90 [3.8-4.0]	3.82 [3.7–3.9]	0.760	0.016	0.367	
Normal	3.85 [3.7-4.0]	3.88 [3.7-4.0]	3.87 [3.8-4.0]				
Overall, B	3.80 [3.7–3.9]	3.89 [3.8-4.0]					
**Pressure to Eat**							
Thin	4.37 [4.2–4.6]^a^	4.18 [4.0-4.4]^ab^	4.27 [4.1–4.4]^x^	< 0.001	0.595	< 0.001	
Normal	3.49 [3.3–3.7]^c^	3.96 [3.8–4.1]^b^	3.72 [3.6–3.8]^y^				
Overall, B	3.93 [3.8–4.1]	4.07 [3.9–4.2]					
**Monitoring**							
Thin	3.97 [3.8–4.2]	3.97 [3.8–4.2]	3.97 [3.8–4.1]	0.574	0.778	0.543	
Normal	4.16 [4.0-4.3]	3.96 [3.8–4.1]	4.06 [3.9–4.2]				
Overall, B	4.07 [3.9–4.2]	3.96 [3.8–4.1]					
**Behavioral Pediatrics Feeding Assessment Scale scores**							
**Picky eaters**							
Thin	21.8 [20.8–22.8]^a^	20.2 [19.2–21.2]^b^	21.0 [20.3–21.7]^x^	< 0.001	0.809	0.001	
Normal	16.6 [15.6–17.5]^d^	18.4 [17.5–19.4]^c^	17.5 [16.8–18.2]^y^				
Overall, B	19.2 [18.49–19.9]	19.3 [18.5–18.6]					
**Toddler general refusal**							
Thin	13.1 [12.3–13.9]^a^	13.3 [12.5–14.1]^a^	13.2 [12.6–13.7]^x^	< 0.001	< 0.001	< 0.001	
Normal	8.9 [8.1–9.6]^b^	12.8 [12.0- 13.5]^a^	10.8 [10.3–11.4]^y^				
Overall, B	11.0 [10.4–11.5]^m^	13.0 [12.5–13.6]^n^					
**Toddler textured food refusal**							
Thin	7.8 [7.3–8.3]^a^	7.0 [6.5–7.4]^b^	7.4 [7.0- 7.7]^x^	0.002	0.294	0.016	
Normal	6.4 [5.9–6.9]^b^	6.8 [6.4–7.3]^b^	6.6 [6.3-7.0]^y^				
Overall, B	7.1 [6.8–7.4]	6.9 [6.5–7.2]					
**Older child refusal**							
Thin	16.3 [15.4–17.1]^a^	15.1 [14.2–15.9]^b^	15.7 [15.1–16.2]^x^	< 0.001	0.392	< 0.001	
Normal	13.2 [12.4–14.0]^c^	15.1 [14.2–15.9]^b^	14.1 [13.6–14.7]^y^				
Overall, B	14.7 [14.2–15.3]	15.1 [14.5–15.6]					
**Total**							
Thin	59.0 [56.6–61.3]^a^	55.5 [53.1–57.8]^b^	57.2 [55.58.9]^x^	< 0.001	0.217	< 0.001	
Normal	45.0 [42.7–47.3]^c^	53.1 [50.8–55.4]^b^	49.1[47.4–50.7]^y^				
Overall, B	52.0 [50.4–53.6]	54.3 [52.6–55.9]					
**Figure Rating Scale**							
**Image of perceived weight of child**							
Thin	2.37 [2.2–2.5]^b^	2.01 [1.8–2.2]^a^	2.19 [2.1–2.3]^x^	< 0.001	< 0.001	0.001	
Normal	3.62 [3.4–3.8]^d^	2.64 [2.5–2.8]^c^	3.13 [3.0- 3.2]^y^				
Overall, B	2.99 [2.9–3.1]^m^	2.32 [2.2–2.4]^n^					
**Image of desired weight of child**							
Thin	4.28 [4.1–4.4]	4.27 [4.1–4.4]	4.28 [4.2–4.4]	0.655	0.811	0.537	
Normal	4.15 [4.0-4.3]	4.41 [4.3–4.6]	4.28 [4.2–4.4]				
Overall, B	4.22 [4.1–4.3]	4.34 [4.2–4.5]					

Values are estimated means [95% confidence intervals]

Values carrying different letters [^abcd^(AXB);^mn^(B), and ^xy^(C)] are significantly different, p < 0.05

*Generalized linear models analysed the interaction between scale scores and variables [nutritional status (A), study center (B) and their interaction (AXB)] after adjusting mother’s age (years), mother’s education (Illiterate, Primary-Middle, high school, University), income (low, middle), child number (n), birth weight (g) with LSD (least significant difference) for subgroup analysis

The mean score for the Perceived Responsibility subscale was found to be lower in those living in Sanlıurfa compared to those in Ankara (p < 0.001) and in those with normal weight compared to thin ones (p = 0.022). An interaction between nutritional status and study center was detected and the perceived responsibilities of thin children living in Ankara had the highest score (p = 0.048, Table 3). The mean score of the Perceived Child Weight subscale was higher in Ankara than that in Şanlıurfa and in normal weight than that in thin cases (p < 0.001,<0.001, respectively. Table 3). Both the Concern about Child Weight and Pressure to Eat subscales were higher in thin cases than in normal weight cases. In addition, Pressure to Eat subscale had the highest point in thin cases from Ankara and the lowest in normal weight from Ankara.

When confounding factors such as mother’s age, mother’s education level, income, count of children, and birth weight-adjusted, Şanlıurfa had higher scores for the Restriction subscale than Ankara. Mothers living in Ankara with thin children were the group that perceived responsibility the most for their children’s nutrition and restricted them the least (p = 0.003, 0.016, respectively). Mothers with thin children from both cities perceived responsibility more, perceived their child’s weight less, were concerned about child’s weight more and pressured their children to eat more (p = 0.010, < 0.001, < 0.001, < 0.001 respectively, Table 3).

### BPFAS scores in groups

Mothers having thin children had higher scores for all BPFAS subscales and total scale than mothers with normal weight children (p < 0.001, Table 3). Only toddler general refusal subscale scores were found to be higher in mothers from Şanlıurfa than those from Ankara Center (p < 0.001, Table 3). All scores showed interaction according to the study centers and nutritional status. Mothers having thin children in Ankara had the highest scores for total BPFAS, picky eaters, toddler textured food refusal, and older child refusal subscales. On the contrary, mothers having normal weight children in Ankara had the lowest scores for all subscales of BPFAS (Table 3).

**Figure Rating Scale**.

Mothers of both thin and normal-weight children in Şanlıurfa rated their children as weaker compared to those in Ankara (p < 0.001). Image of perceived child weight scores were the lowest in thin children from Şanlıurfa and the highest in normal-weight children from Ankara (p < 0.001). The desired body image was similar in both cities and both nutritional status (Table 3).

### Correlations between Subscales

**Fig. 1:**
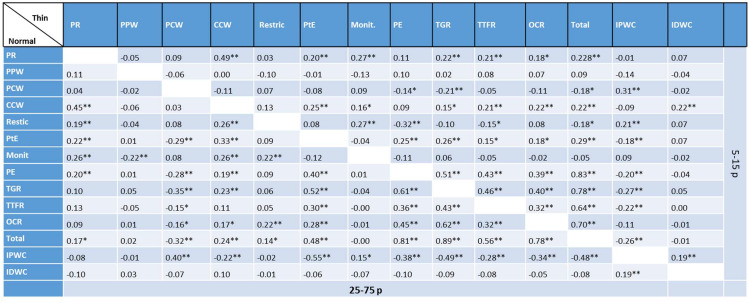
Spearman’s rho correlations between subscales of Child Feeding Questionnaire and Behavioral Pediatrics Feeding Assessment Scale, and figure rating scales in thin and normal weight children [* Correlation is significant at the 0.05 level (2-tailed); ** Correlation is significant at the 0.01 level (2-tailed); PR: Perceived Responsibility; PPW: Perceived Parent Weight; PCW: Perceived Child Weight; CCW: Concern about Child Weight; Restric: Restriction; PtE: Pressure to Eat; Monit: Monitoring; PE: Picky eaters; TGR: Toddler general refusal; TTFR: Toddler Textured Food Refusal; OCR: Older Child Refusal; IPWC: Image of Perceived Weight of Child; IDWC: Image of desired weight of child; CFQ: Child Feeding Questionnaire; BPFAS: Behavioral Pediatrics Feeding Assessment Scale]

There is an association between Perceived Responsibility and Concern about Child Weight in both thin and normal weight children (r = 0.49, p < 0.01 and r = 0.45, p < 0.01, Fig. 1). In the thin and normal weight group Toddler general refusal scores were related to Toddler textured food refusal (r = 0.46 and r = 0.43) and Older child refusal scores (r = 0.40 and r = 0.62). Also, in thin and normal weight groups, picky eaters scores were correlated with Toddler general refusal (r = 0.51 and r = 0.61), Toddler textured food refusal (r = 0.43 and r = 0.36), Older child refusal scores (r = 0.39 and r = 0.45).

When correlation coefficients more than │0.40│ were considered, the image of the desired weight of the child in normal-weight children positively correlated with Perceived child weight (r = 0.40) and negatively related with Pressure to eat (r=-0.55), and Toddler general refusal (r=-0.48). In normal-weight children all correlations for the image of desired weight of child were below 0.40. No interaction having r> │0.40│ was detected for perceived parent weight, restriction, monitoring, and image of the desired weight of child with other subscales (Fig. 1).

## Discussion

In our study, regional differences were detected for the duration of breastfeeding, and time to introduction of complementary food. Mothers in Ankara had a longer duration of breastfeeding and lower percentages for the delayed introduction of complementary food. In addition, parental feeding practices were associated with the weight of children depending on demographic and sociocultural differences.

Mothers of thin children living in Ankara had the highest level of perceived responsibility for their children’s nutrition, concerned about child weight, and pressured them to eat more. The lowest levels were found in mothers of normal weight children from Ankara for concern about child weight, pressure to eat subscales and from Şanlıurfa for the perceived responsibility subscale. The changing ideal body image, especially in western countries, increases body image dissatisfaction and has been a driving force for weakening. Weakness is associated with socioeconomic status, especially in western culture, and is perceived to increase social acceptance [27]. In contrast, in non-western cultures, plumpness is often associated with higher social status, higher fertility, and being more attractive. Chubby children are accepted as healthy children and there is social pressure to maintain a heavier weight in low-income societies [28–31]. Therefore, perceived child weight might be lower in both the thin and normal weight groups in Şanlıurfa than in Ankara. Due to the fact that mothers from Şanlıurfa have more children, they can give less time to feed each child and they are freer for selectivity of healthy diet in eastern-southeastern provinces compared to western provinces. This situation is thought to be related to less perceived responsibility for child nutrition, less pressure to eat, and less concern about child’s weight in mothers from Şanlıurfa.

The mothers of thin children perceived the weight of their children lower and were concerned more about it. They felt more responsible for feeding their children and pressured them to eat more frequently compared to normal-weight children’s mothers in both cities. The relationship between body weight/ perceived body weight and this feeding behavior have been shown in many studies. Bangchum et al. showed that mothers were more concerned about child nutrition and used more pressuring strategies if children are underweight or mothers perceived their children as underweight [32]. A similar approach across perceived child weight was found in other studies [33, 34]. Maternal perception of the child as thin causes pressure on them to eat more due to the desire to have a heavier child. They may think that thinner children are biologically weaker and unhealthy, and therefore their growth and development will stall. However, this pressure may result in the child consuming healthy and also unhealthy foods more and gaining weight. Conversely, pressure to eat can trigger food anxiety, food avoidance, fussy eating and picky eating resulting in lower child weight [35, 36]. Therefore, they should pay particular attention to promoting a balanced diet with healthy foods.

No significant difference was found between cities and nutritional status for perceived parent weight, restriction, and monitoring. The monitoring subscale measures how often mothers monitor the consumption of junk foods, candies, and snacks of their children. Monitoring was also not significantly associated with maternal perception and child weight in previous studies [33, 37]. Preschool-age children’s mothers may prefer to restrict or change the food environment around their children instead of monitoring. One factor affecting childhood weight is suggested to be the parents’ obesity. Studies have shown significant correlations between children’s BMIs and their parent’s BMIs [38]. However, the mother’s BMI was not calculated in our study. In addition, we investigated only thin children and normal weight children. Mothers with thin children may support them to gain weight with healthy foods by restricting unhealthy and satiating foods such as sugary and high-fat junk foods. On the contrary, food restriction may be triggering more eating with the opposite effect. Studies showed that when the parents restricted more, children developed an increased preference for the foods [39]. Bauer et al. reported negative associations between maternal restriction of food amount at 21 months and child eating at 27 months [40]. Birch et al. observed that eating in the absence of hunger was more common among girls aged 5–9 whose mothers reported high levels of restriction [41]. Another study supported these results by showing a positive association between parental restriction of food for health reasons and the use of food as a reward and more eating behavior in 35 preschool-aged children, 5 to 7 years [42]. As we determined in our study, children should be supported in the consumption of healthy foods instead of making restrictions.

For all sub-dimensions of BPFAS, the lowest scores were found in normal-weight children from Ankara and the highest scores were found in low-weight children from Ankara. Picky eating, toddler, and older childhood food refusal were more common in thinner children in both cities. Although many studies have not found a correlation between food refusal and body weight [43], it is necessary to conduct further studies to determine the long-term effects of food refusal. The reasons for food refusal are not fully known but several biological, psychological, environmental, medical, and behavioral factors and experiences gained from contact with foods in early childhood have been identified that cause this situation. Younger mother age, lower maternal education status, lower-income, increased number of children at home, lower birth weight, having a family member or another child with food refusal at home, inappropriate feeding techniques might be associated with food refusal and other eating problems [44–48].

General food refusal in toddler was more common in Şanlıurfa than in Ankara. Shorter breastfeeding duration, delayed introduction of complementary feeding and solid foods, high percentages of formula usage, and insufficient count of the meal on a day were found in Şanlıurfa. All these factors may result in delayed stimulation of suck/swallow reflexes in the brain stem of children, decreased variety and amount at mealtimes. In the end, these factors may be responsible for general refusal in toddlers from Şanlıurfa. By developing strategies for the solution of mothers’ breastfeeding problems and supporting mothers in breastfeeding, both eating problems of children will be prevented and the health outcomes of children will be improved potentially.

Thin children with textured food refusal in the toddler period also refused general food in this period. Although toddler food refusal usually disappears spontaneously over time in childhood, especially in severe cases, if not diagnosed early and precautions are not taken, it may affect the way of eating and eating habits of the child in the future life [43]. In addition, both textured and general food refusal in toddlers and older childhood are related to picky eaters. As toddler general refusal scores increase, pressure to eat scores increase also in the normal weight group (r = 0.52). To avoid a lack of a balanced diet and reduced important nutrient intake, families and clinicians should be careful in terms of food refusal and support the child’s healthy growth and development by preventing it with an appropriate approach.

### Strengths and limitations

The main strengths of this study are that it was applied to two cities with different socio-demographic and cultural characteristics and compared the two groups by dividing the patients as thin and normal weight. The use of two different advanced tools to scale both children’s eating behaviors and parents’ feeding attitudes contributed to a more accurate and objective determination of differences depending on body weight between different cities. Study results can moderate the development of specific regional strategies for solving these problems.

The main limitation of this study is the use of a self-reported measurement. Although self-reporting is a successful method in measuring participants’ orientations, it is based on subjective data. However, considering to evaluation of sub-dimensions of the questionnaires and their strong correlation with each other, it can be thought that participants’ biases are prevented as much as possible.

## Conclusion

This study demonstrated that child eating behaviors and parental feeding practices were associated with the weight of children depending on demographic and sociocultural differences among children aged 2–7 years. It may be beneficial to organize local preventive educational programs on child feeding for families and to evaluate the effect of behavioral changes on children’s eating habits and their body weights after these trainings in future studies.

### Electronic supplementary material

Below is the link to the electronic supplementary material.

**Supplementary Material 1:** Strobe Statement of the study

## Data Availability

Data available on request from the corresponding author (siyalcin@hacettepe.edu.tr).
